# Tight Junction Claudins and Occludin Are Differentially Regulated and Expressed in Genomically Defined Subsets of Colon Cancer

**DOI:** 10.3390/cimb45110545

**Published:** 2023-10-28

**Authors:** Ioannis A. Voutsadakis

**Affiliations:** 1 Algoma District Cancer Program, Sault Area Hospital, Sault Ste Marie, ON P6B 0A8, Canada; ivoutsadakis@yahoo.com or ivoutsadakis@nosm.ca; 2 Division of Clinical Sciences, Section of Internal Medicine, Northern Ontario School of Medicine, Sudbury, ON P3E 2C6, Canada

**Keywords:** claudins, occludin, consensus molecular subtypes, bowel cancer, genomics

## Abstract

Metastatic colon cancer remains incurable despite improvements in survival outcomes. New therapies based on the discovery of colon cancer genomic subsets could improve outcomes. Colon cancers from genomic studies with publicly available data were examined to define the expression and regulation of the major tight junction proteins claudins and occludin in genomic groups. Putative regulations of the promoters of tight junction genes by colon-cancer-deregulated pathways were evaluated in silico. The effect of claudin mRNA expression levels on survival of colon cancer patients was examined. Common mutations in colon-cancer-related genes showed variable prevalence in genomically identified groups. Claudin genes were rarely mutated in colon cancer patients. Genomically identified groups of colon cancer displayed distinct regulation of claudins and occludin at the mRNA level. Claudin gene promoters possessed clustered sites of binding sequences for transcription factors TCF4 and SMADs, consistent with a key regulatory role of the WNT and TGFβ pathways in their expression. Although an effect of claudin mRNA expression on survival of colon cancer patients as a whole was not prominent, survival of genomic subsets was significantly influenced by claudin mRNA expression. mRNA expression of the main tight junction genes showed differential regulation in various genomically defined subgroups of colon cancer. These data pinpoint a distinct role of claudins and pathways that regulate them in these subgroups and suggest that subgroups of colon cancer should be considered in future efforts to therapeutically target claudins.

## 1. Introduction

Colorectal cancer is the major cause of gastrointestinal cancer morbidity and mortality, especially in Western populations, where gastric and hepatocellular cancers have a lower prevalence than in Asian populations [1]. Over 1.8 million patients were diagnosed with colorectal cancer worldwide in 2018, and almost half of these patients died from the disease [2].

Colorectal cancers are heterogeneous and can be classified in different subsets derived from genomic studies [3]. Genomic classifications of colorectal cancer attempt to capture the heterogeneity of the disease at the genomic level based on mRNA expressions. The Cancer Genome Atlas (TCGA) classifies colorectal cancers in three main genomic categories: with microsatellite instability (MSI), with chromosomal instability (CIN), and genomically stable (GS) [4]. An international consortium categorized colorectal cancers into four consensus molecular subtypes (CMS), called CMS1 to CMS4, based on a consensus from six previous molecular classifications [5]. The CMS1 category corresponds to the subset with MSI. CMS2, which is the most extensive group, shows CIN and increased WNT pathway signaling. CMS3 is characterized by metabolic deregulation, and CMS4 is characterized by TGFβ pathway activation and invasiveness. A fifth group contains colorectal cancers with mixed features of two or more of the other groups [5]. The consensus molecular classification has been used by other investigators, including a recent cohort study published by an international consortium, providing an opportunity for analyses based on CMS categories [6]. Categorization of colorectal cancers according to key genomic properties and identification of the related main pathways underscoring their pathogenesis may aid in the discovery of additional key cancer-associated processes that would otherwise be less evident in whole cohorts of colorectal cancers. In addition, genomic classes may help unveil therapeutic vulnerabilities and guide treatment directions.

Tight junctions safeguard the integrity of the colonic epithelial sheath and regulate permeability, playing important roles in bowel physiology [7]. Tight junctions are located at the upper-most domain of the basolateral plasma membrane and represent the most apical junctional contact between neighboring epithelial cells. The family of claudin proteins and occludin belonging to the MARVEL (MAL and related proteins for vesicle trafficking and membrane link) family of proteins form the transmembrane links interacting with homologous proteins of the neighboring cell. The other type of epithelial cell junctions, adherens junctions, are located more basal to the tight junctions in the basolateral plasma membrane. Adherens junctions are created with homotypic interactions of E-cadherin. Besides its adhesion role, E-cadherin plays a signaling role in the WNT/β-catenin signal transduction pathway, which is important in the pathogenesis of colorectal cancer [8]. In cellular conditions of low WNT activation, β-catenin remains cytoplasmic and interacts with E-cadherin in the plasma membrane or becomes phosphorylated and subsequently ubiquitinated and degraded in the proteasome through the action of a destruction complex that contains tumor suppressors APC and axin 2 as scaffolds, kinases CK1 and GSK3, and ubiquitin ligase β-TrCP [8]. When WNT receptors are ligated by ligands of the Frizzled family, components of the destruction complex are sequestered in the receptor and β-catenin is freed to enter the nucleus and act as a transcription factor in collaboration with transcription factor TCF. TCF/β-catenin plays a key role in the epithelial-to-mesenchymal transition (EMT) through induction of the transcriptional regulator Snail.

EMT is a process that cancer cells have co-opted from embryonic development, and it is involved in cell motility and metastatic dissemination through loss of intercellular adhesions [9]. During EMT, epithelial cancer cells acquire mesenchymal morphology and modify protein expression through the activity of core EMT transcription regulators, such as ZEB1 and ZEB2, Snail and Snail2 (also called Slug), and Twist1 [10]. Core EMT factors are regulated by signaling pathways altered in cancer, such as TGFβ and WNT [8]. The activity of core EMT factors leads to downregulation of adhesion molecules, including claudins. Downregulation of adhesion molecules and dissolution of intercellular adhesions are defining features of EMT, which creates mobile mesenchymal cells [11]. The genes encoding for claudins are, in general, not altered in subsets of cancers that display suppression of their protein expression, such as claudin-low breast cancers [12]. Instead, suppression of their mRNA expression and post-translational deregulation are observed, resulting in their decreased protein expression during the EMT process.

Claudin regulation has not been reported in detail in colorectal cancer as it pertains to genomic subsets. In the current investigation, the role of deregulation of several claudins in genomic subsets of colorectal cancers was explored in conjunction with molecular alterations present in each subtype. The potential direct or indirect regulations of their expression by signaling pathways and core EMT factors were also examined.

## 2. Materials and Methods

Two genomic studies of colorectal cancer with publicly available data, the Cancer Genome Atlas (TCGA) colorectal study and the Sidra-LUMC AC-ICAM international cohort study, were used in the current investigation [4,6]. The TCGA colorectal cancer cohort contained 594 patients, among which 335 had cancers located in the colon and were assigned to one of 3 genomic categories: chromosomal unstable (CIN), microsatellite unstable (MSI), and genomically stable (GS). Rectal cancers were excluded from the current analysis. TCGA used a whole-exome sequencing platform [4]. Single-nucleotide mutation calling was performed with the use of various pipelines [13]. Analysis of copy number alterations in TCGA was performed with the GISTIC (genomic identification of significant targets in cancer) algorithm [14]. The GISTIC algorithm assigns a score of 2 or above to genes with putative amplifications, while genes with a score of −2 or below are considered homodeleted [14]. RNA expression was normalized with the RSEM algorithm, and results were presented as log RNA sequences in reads per kilobase million (RPKM) [15].

The Sidra-LUMC AC-ICAM study with 348 colon patients also used whole-exome sequencing [6]. Mutation calling in this cohort was made with the mutect (v.1.1.7) algorithm. The study categorized patients into one of 5 consensus molecular subgroups (CMS1 to CMS4 and a mixed group) according to the schema proposed by a collaborative consortium of investigators [5].

Analyses from TCGA and the Sidra-LUMC AC-ICAM studies were performed in the cBioCancer Genomics Portal (cBioportal, http://www.cbioportal.org, accessed on 11 August 2023), a genomics site maintained by MSKCC and other academic institutions [16,17]. Genomic cohorts included in cBioportal can be analyzed at the level of individual samples, and individual genes contained in the source studies can be analyzed to identify point mutations; copy number alterations; and structural gene alterations, such as fusions. The two studies of interest were selected sequentially from the master list of the cBioportal site for further analysis. Sample subsets of interest were selected in the main board of the specific study through the custom selection function. Mutations or other genomic alterations were then quantified in the given subset of interest. Subgroups with or without alterations of interest were further analyzed through the download function and the altered or unaltered samples tab provided in the cBioportal site. For calculations not directly provided in cBioportal, the datasets were copied in an Excel spreadsheet (Microsoft Corp., Redmond, WA, USA) to perform the required analyses.

Sequences of gene promoters were identified in the Eukaryotic Promoter Database (EPD, www.epd.expasy.org/epd, accessed on 16 August 2023) [18]. This database, hosted by the Swiss Institute of Bioinformatics resources site, provides accurate transcription start sites from several species, including humans. Parameters used for motif search were area from −1000 to 100 base pairs from the transcription start site and cut-off p-value of 0.001. Binding motifs of transcription factors of interest were retrieved through the open-access JASPAR CORE 2018 vertebrates database [19]. The database contains curated binding motifs of transcription factors presented as position frequency matrices (PFMs). Except in cases where a given transcription factor has multiple preferred binding sites, the JASPAR database is nonredundant, containing one matrix profile per transcription factor per species [19].

Survival analysis according to tight junction protein mRNA expression levels was performed on the online tool Kaplan–Meier plotter (www.kmplot.com, accessed on 31 August 2023) [20]. This tool uses publicly available cohorts of cancer patients with genomic and clinical data deposited in the European Genome–Phenome Archive (ega-archive.org, accessed on 31 August 2023), the Gene Expression Omnibus (GEO) database (ncbi.nlm.nih.gov/geo, accessed on 31 August 2023) of the National Center for Biotechnology Information, and TCGA. The optimally performing quartiles of mRNA expressions were selected for the survival analysis.

Statistical comparisons of categorical data were carried out with the Fisher exact test or the χ^2^ test, and analyses of continuous data were performed using the *t* test or ANOVA. The log-rank test was used to compare Kaplan–Meier survival curves. All statistical comparisons were considered significant if *p* < 0.05. Corrections for multiple comparisons were performed with the Bonferroni procedure.

## 3. Results

**Figure 1:**
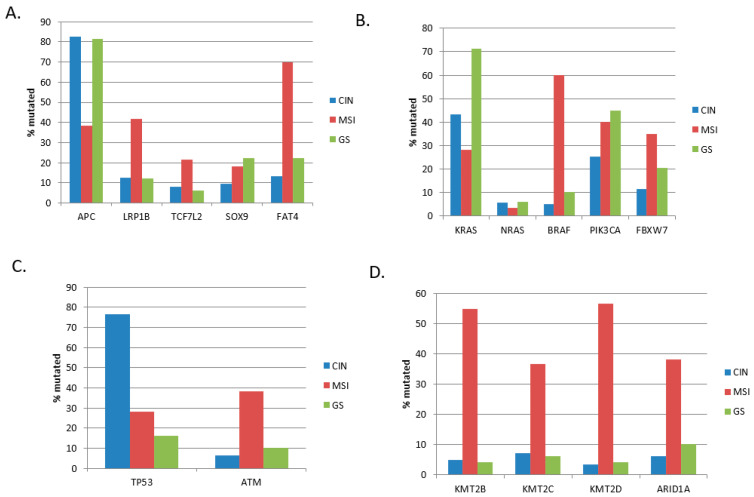
Prevalence of frequently mutated colorectal cancer genes according to colon cancer subtypes. (**A**) Genes of the WNT/β-catenin pathway, (**B**) KRAS/BRAF and KRAS/PI3K pathway genes, (**C**) most frequently mutated DNA damage response genes *TP53* and *ATM*, (**D**) epigenetic modifiers. CIN: group of cancers with chromosomal instability, MSI: group of cancers with microsatellite instability, GS: group of genomically stable colon cancers. Data are from the Cancer Genome Atlas (TCGA).

The colorectal cancer cohort from TCGA contained 335 colon cancers divided into three genomic categories. The most extensive group with 67.5% of cases (226 samples) consisted of colon cancers with chromosome instability (CIN). A second group with 17.9% of cases (60 samples) was characterized by microsatellite instability (MSI), and the third group with 14.6% of cases (49 samples) was genomically stable (GS). The three groups showed significant differences in the prevalence of common colon cancer mutations (Figure 1). The prevalence of tumor suppressor *APC* mutations was higher in CIN and GS colon cancers, where these mutations were present in over 80% of cases, while they were less frequent in MSI colon cancers (38.3% of cases). In contrast, mutations in other genes encoding for WNT pathway proteins, including *LRP1B*, *TCF7L2*, and the gene encoding for the atypical cadherin *FAT4*, were more prevalent in MSI tumors (Figure 1A). Oncogene *KRAS* mutations were most frequently observed in GS colon cancers (71.4%) and less frequently in CIN cancers (43.4%) and in the MSI group (28.3%, χ^2^ test *p* = 0.00003). The latter group was characterized by a high frequency of *BRAF* mutations instead (χ^2^ test *p* < 0.00001, Figure 1B). Tumor suppressor p53 gene mutations were significantly more prevalent in CIN colon cancers (76.5%) compared to MSI (28.3%) and GS (16.3%) cancers (χ^2^ test *p* = 0.001, Figure 1C). In contrast, mutations in another gene involved in the DNA damage response, *ATM,* were more common in the MSI group. Mutations in epigenetic modifier enzymes KMT2B, KMT2C, KMT2D, and ARID1A were more prevalent in MSI cancers, where the majority possessed KMT2B and KMT2D mutations and over one-third had KMT2C and ARID1A mutations (χ^2^ test *p* = 0.001, Figure 1D).

**Figure 2:**
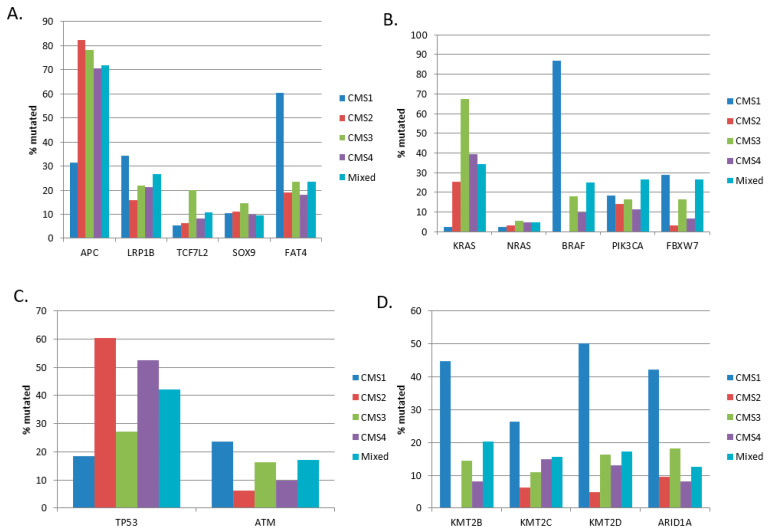
Prevalence of frequently mutated colorectal cancer genes according to colon cancer subtypes. (**A**) Genes of the WNT/β-catenin pathway, (**B**) KRAS/BRAF and KRAS/PI3K pathway genes, (**C**) most frequently mutated DNA damage response genes *TP53* and *ATM*, (**D**) epigenetic modifiers. CMS: consensus molecular subtype. Data are from the Sidra-LUMC AC-ICAM cohort.

In the Sidra-LUMC AC-ICAM cohort, CMS1 (38 cases) containing MSI colorectal cancers presented lower rates of mutations in *APC*, *KRAS,* and *TP53* than other groups and higher rates of mutations in *BRAF* and epigenetic modifiers (Figure 2). The three other groups, CMS2, CMS3, and CMS4 (63, 55, and 61 cases, respectively), had more similar rates of mutations, with the notable exception of *KRAS* and *TP53* mutations (Figure 2B,C). *KRAS* mutations were more prevalent in CMS3 (67.3%) compared to the CMS2 and CMS4 groups (25.4% and 39.3%, respectively, χ^2^ test *p* = 0.00001, Figure 2B). Inversely, *TP53* mutations were more frequent in CMS2 and CMS4 (60.3% and 52.5%, respectively) than in CMS3 (27.3%, Figure 2C).

Mutations in the genes encoding for claudins 1 to 7 were present in only a small number of colon cancer cases in TCGA. The most frequently mutated claudin genes in the TCGA cohort with 2.1% of cases each were the genes for claudins 10 and 18, *CLDN10* and *CLDN18*. In the Sidra-LUMC cohort, *CLDN17* was the most frequently mutated claudin, being present in 2.8% of cases. Similar to the TCGA cohort, mutations in claudins 1 to 7 were rare in the Sidra-LUMC AC-ICAM cohort, with the most prevalent mutations being in *CLDN6* in 5 cases of the 281 sequenced samples (1.8%) and the rest showing mutations in 0 to 2 cases.

**Figure 3:**
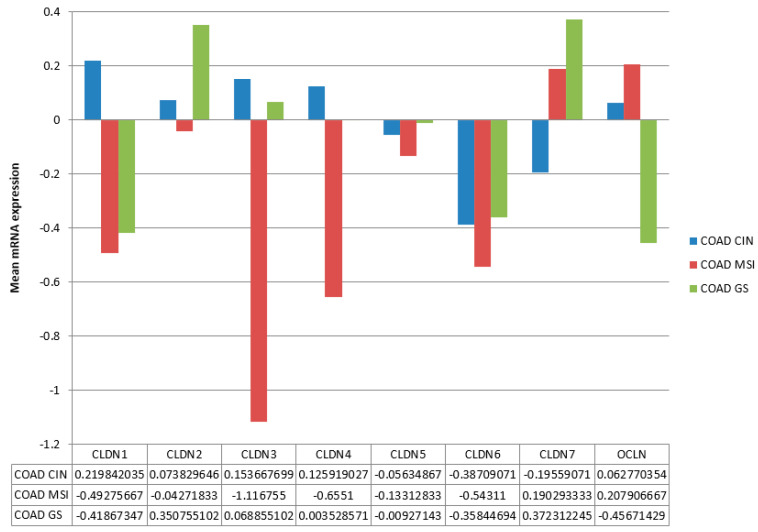
mRNA expression of the main claudins and occludin in subtypes of colon cancer. CIN: group of cancers with chromosomal instability, MSI: group of cancers with microsatellite instability, GS: group of genomically stable colon cancers. Data are from the Cancer Genome Atlas (TCGA).

Expression of claudins 1 to 7 and occludin varied in molecular types of colon cancers in TCGA (Figure 3). The most extensive group of colon cancers, those with chromosomal instability (CIN), was characterized by modest upregulation of expression of claudin 1 and downregulation of claudin 7. Cancers with MSI were characterized by downregulation of claudin 1, claudin 3, and claudin 4 and upregulation of claudin 7 and occludin. Genomically stable colon cancers were characterized by downregulation of claudin 1 and occludin and upregulation of claudin 2 and claudin 7 (Figure 3). All three types showed downregulation of claudin 6. Differences in expression were statistically significant for all claudins except claudin 5 and claudin 6 and also significant for occludin expression after adjustment for multiple comparisons (ANOVA *p* < 0.005).

Also similar to the TCGA cohort, the expressions of claudins were divergent in the genomic CMS groups in the Sidra-LUMC AC-ICAM cohort. CMS1 was characterized by suppression of claudin 1, claudin 3, and claudin 4 and upregulation of occludin mRNA, consistent with the corresponding MSI group of TCGA. CMS2, on the other hand, displayed upregulation of claudin 1, claudin 3, and claudin 4 mRNAs, trends also observed in the overlapping CIN group of TCGA. CMS3 cases were characterized by suppression of claudin 1 and upregulation of claudin 2 and claudin 7 mRNAs, consistent with the GS group of TCGA. CMS4 was characterized by suppression of claudin 2, claudin 3, claudin 7, and occludin and upregulation of claudin 5 mRNAs, not aligning with any of the three TCGA genomic groups or with CMS2 despite the similarities in the prevalence of common colon cancer mutations. Differences between CMS groups in expression were statistically significant for all claudins and occludin after adjustment for multiple comparisons (ANOVA *p* < 0.005).

Examination of claudin promoters listed in EPD for the presence of binding sites for transcription factors of pathways involved in colorectal carcinogenesis disclosed that all claudins examined possessed clustered binding sites for SMAD transcription regulators and several also possessed clustered binding sites for the WNT/β-catenin pathway transcription factor TCF4 (Table 1). This suggests that claudin promoters are regulated by the TGFβ and the WNT pathways. Occludin had clustered binding sites for TCF4 but not for SMAD. Both claudins and occludin promoters possessed binding sites for EMT core transcription factors. In contrast, p53 and the colorectal-specific homeobox transcription factor CDX2 showed no clustered binding sites in any of the examined tight junction proteins. Transcription factor SOX9, which is induced by WNT signaling but is also a suppressor of β-catenin, had no clustered sites in the claudins examined or in occludin. MYC and transcription factor AP-1 (FOS-JUN heterodimer), which is activated downstream of the KRAS/MAPK pathway, displayed clustered binding sites in promoters of CLDN7 and *OCLN* genes and *CLDN4* gene, respectively.

**Figure 4:**
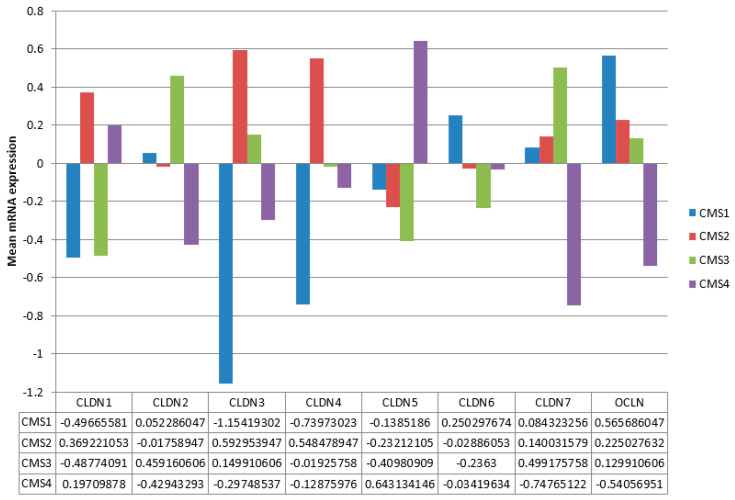
mRNA expression of the main claudins and occludin in subtypes of colon cancer. CMS: consensus molecular subtype. Data are from the Sidra-LUMC AC-ICAM cohort.

**Figure 5:**
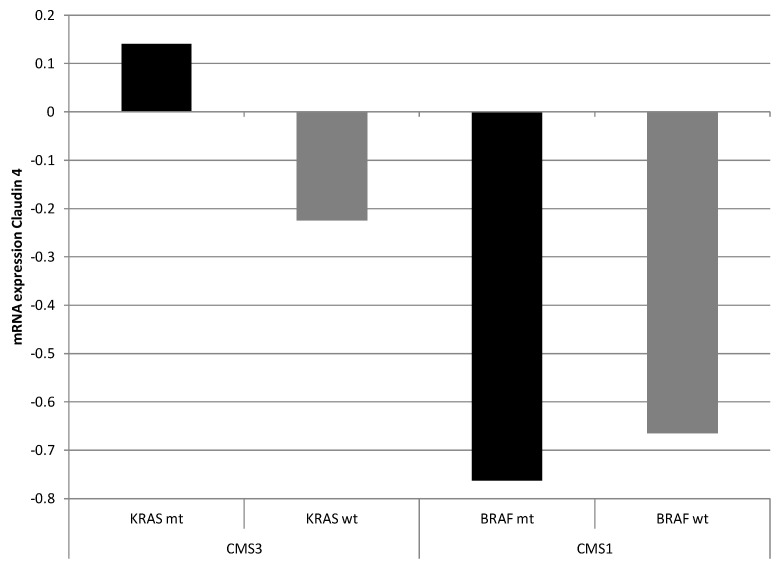
mRNA expression of claudin 4 in CMS3 colon cancers with (black bar) and without (gray bar) *KRAS* mutations (left) and in CMS1 colon cancers with (black bar) and without (gray bar) *BRAF* mutations (right). CMS: consensus molecular subtype. Data are from the Sidra-LUMC AC-ICAM cohort.

Given the putative regulation of *CLDN4* by AP-1 through AP-1-binding sites in its promoter, mRNA expressions of CLDN4 within molecular subsets of colorectal cancers were examined taking into consideration the presence of mutations in the upstream regulators KRAS and BRAF. In the Sidra-LUMC AC-ICAM cohort, the CMS3 subset of colon cancers, which possessed the highest prevalence of *KRAS* mutations, displayed a neutral expression of claudin 4 as a whole and did not show upregulation or suppression compared to the entire cohort (Figure 4). CMS3 cancers with *KRAS* mutations presented a slight upregulation of claudin 4, while CMS3 cancers with wild-type *KRAS* displayed suppressed expression of claudin 4 (two-tailed *t* test *p* < 0.05, Figure 5, left). On the other hand, CMS1 cancers, which possessed the highest prevalence of *BRAF* mutations, presented a downregulated expression of claudin 4 mRNA, independently of the presence or absence of *BRAF* mutations (two-tailed *t* test *p* > 0.05, Figure 5, right).

**Figure 6:**
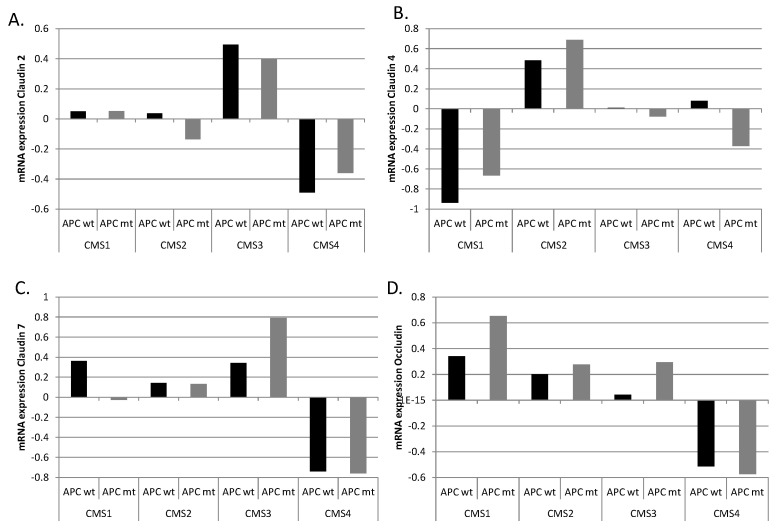
mRNA expression of (**A**) claudin 2, (**B**) claudin 4, (**C**) claudin 7, and (**D**) occludin in subtypes of colon cancer with (gray bars) or without (black bars) *APC* mutations. CMS: consensus molecular subtype. Data are from the Sidra-LUMC AC-ICAM cohort.

The presence of APC mutations upregulates WNT/β-catenin signaling, resulting in increased activity of TCF4 transcription. Clusters of TCF4 binding sites are present in promoters of the *CLDN2*, *CLDN4*, *CLDN7,* and *OCLN* genes, and these genes could thus be upregulated in cancers with *APC* mutations. However, no consistent upregulation was observed in mRNA expression of these junction proteins in *APC*-mutated cancers, suggesting a more complex regulation with inputs from other transcription cascades possibly contributing to the final mRNA output (Figure 6). In a few instances, including expression of claudin 7 in CMS3 colon cancers and expression of occludin in CMS1 and CMS3 colon cancers, upregulation was observed in *APC*-mutated cases, implying a central role of the WNT/β-catenin cascade in these cases.

**Figure 7:**
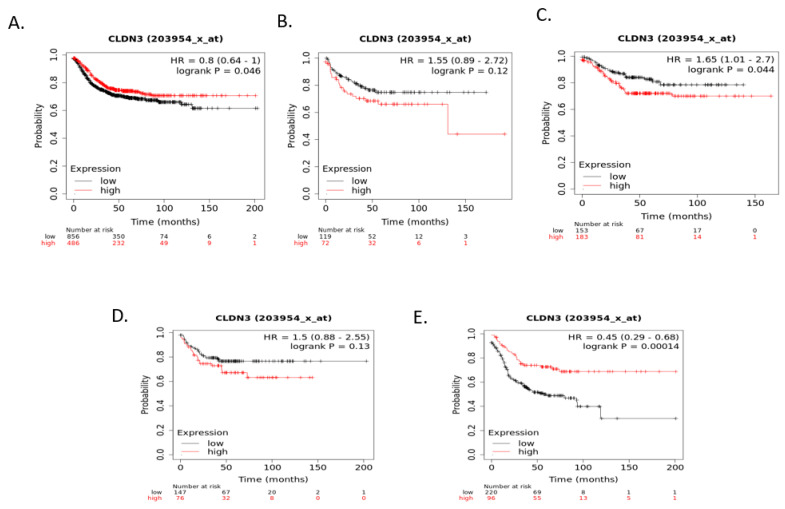
RFS of colorectal cancer patients according to claudin 3 mRNA expression above or below the median. (**A**) All (n = 1342), (**B**) CMS1 (n = 191), (**C**) CMS2 (n = 336), (**D**) CMS3 (n = 221), (**E**) CMS4 (n = 316). Data were generated with the Kaplan–Meier plotter.

**Figure 8:**
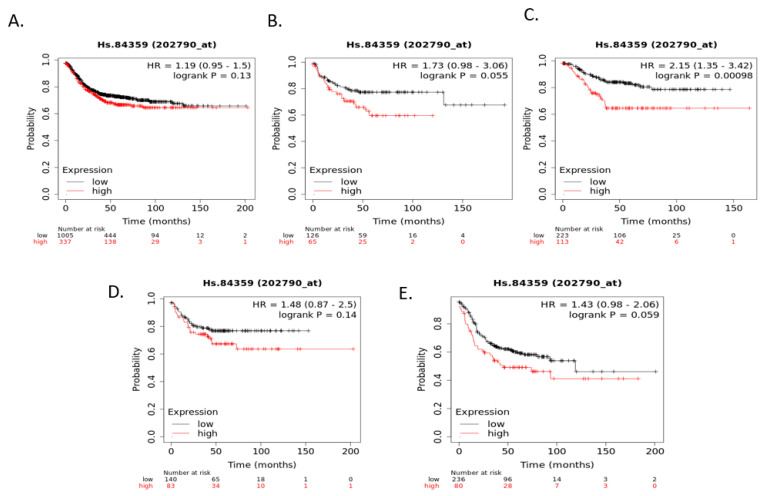
RFS of colorectal cancer patients according to claudin 7 mRNA expression above or below the median. (**A**) All (n = 1342), (**B**) CMS1 (n = 191), (**C**) CMS2 (n = 336), (**D**) CMS3 (n = 223), (**E**) CMS4 (n = 316). Data were generated with the Kaplan–Meier plotter.

**Figure 9:**
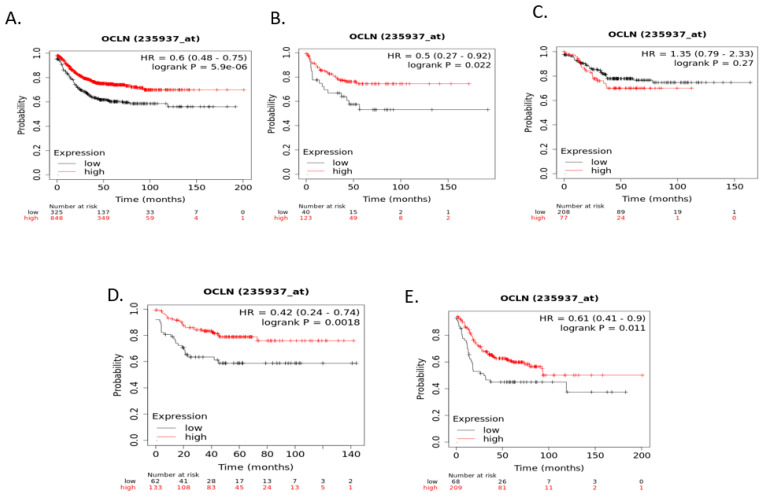
RFS of colorectal cancer patients according to occludin mRNA expression above or below the median. (**A**) All (n = 1173), (**B**) CMS1 (n = 163), (**C**) CMS2 (n = 285), (**D**) CMS3 (n = 195), (**E**) CMS4 (n = 277). Data were generated with the Kaplan–Meier plotter.

Relapse-free survival (RFS) of colorectal cancer patients according to claudin 1, claudin 2, or claudin 4 mRNA expression was not significantly different (after correction for multiple comparisons) when colorectal cancers were examined as a whole or according to CMS groups Although RFS of cancer patients according to claudin 3 expression independently of CMS groups and within CMS1, CMS2, and CMS4 were not significantly different, RFS of CMS4 patients with low claudin 3 expression was significantly worse than that of counterparts with high claudin 3 expression (Figure 7). Claudin 7 mRNA expression was a predictor of RFS in patients with CMS2 colorectal cancers, with patients with low expression presenting a better RFS. In contrast, no significant differences were observed in the whole group and in the other CMS groups (Figure 8). Occludin mRNA expression was a significant predictor of RFS in the whole group of colorectal cancers, with patients with suppressed expression displaying worse RFS. This difference was mostly driven by the CMS3 group, which had significantly worse RFS when occludin was suppressed (Figure 9).

## 4. Discussion

Proteins of the claudin family are among the structural components that play a part in the formation of tight junctions in the upper section of the lateral surface membrane of epithelial cells [21]. Tight junctions participate in the epithelial barrier integrity of the gastrointestinal tract and regulate selective permeability, with both functions being important for maintenance of gut homeostasis [21]. Claudins, together with proteins of the MARVEL family, such as occludin, and junctional adhesion molecules (JAMs), are transmembrane anchors of the tight junctions and are connected to the actin cytoskeleton through intermediate proteins, such as the zona occludens proteins 1, 2, and 3 (ZO-1, ZO-2, and ZO-3) in the junctional plaque [22]. A total of 28 mammalian claudin family members have been described and have variable expression in normal tissues [21]. Claudins 1, 2, 3, 4, and 7 are all expressed in the epithelium of the large bowel. Tissue-specific expression of members of the family is defined by regulation through tissue-specific transcription factors. For example, in the colon, homeobox transcription factor CDX2 regulates *CLDN1* and *CLDN2*, which are upregulated when the WNT pathway is overactivated [23,24]. In normal colon, claudin 1, encoded by gene *CLDN1*, shows a diffuse apical cell pattern of expression, while claudin 2, encoded by gene *CLDN2*, is expressed in undifferentiated crypt cells [25].

The epithelial-to-mesenchymal transition (EMT) is a physiologic process in embryonic development and in adult tissue repair after injuries. During physiologic EMT, epithelial cells lose intercellular adhesions, acquire mesenchymal morphology, and mobilize to participate in organogenesis or to fill areas produced by traumatic injuries [9]. In cancer, EMT programs are activated by signaling cascades associated with carcinogenesis and, similar to the physiologic EMT programs, promote motile cells with metastatic properties [9]. During EMT of cancer, similar to the physiologic counterparts, cancer cells downregulate cellular adhesions, including tight junctions, and acquire fibroblast-like features. This allows detachment from neighboring cells in the tumor mass and infiltration through tissues beyond the primary tumor location. This first step of EMT may be observed in histologic sections as tumor budding [26]. Next, mobile cells infiltrate the vasculature and travel to seed metastatic sites. In those sites, metastatic cells undergo a process reverse to EMT, called mesenchymal-to-epithelial transition (MET), reacquiring epithelial morphology and intercellular adhesions. EMT and MET are together called epithelial-to-mesenchymal plasticity (EMP), emphasizing the fluid state of the cells participating in the two processes. Cancer cells undergoing EMT also possess stem cell properties, empowering them to generate new cells that can populate tumor masses in the metastatic locations [27,28]. The metastatic potential of cancer cells during the metastatic process empowered by EMP involves resolution and reforming of intercellular junctions. Thus, tight junction proteins, such as claudins and occludin, need to be appropriately regulated in an oscillating manner as part of the EMP programs which require dissolution of junctions in the primary site and re-forming at the metastatic site [29]. Nevertheless, capturing the key influence of tight junction proteins on the metastatic process using bulk genomics is challenging. Claudins present infrequent genetic lesions, such as mutations or copy number alterations in cancers. Instead, tight junction upregulation or downregulation relies on transcriptional and post-transcriptional regulations [30]. In addition, the process of EMT in cancer allows the creation of intermediate cell states that have undergone only partial mesenchymal transformation, thus retaining some epithelial characteristics. These intermediate forms may maintain partial adhesions with neighboring cells and can mobilize in clusters instead of individually [31]. The presence of multiple claudins in the human genome is witness to the importance of these proteins in physiology. Overlap of the physiologic roles of paralogous members of the claudin family may decrease the severity of phenotypes observed in the case of defects in individual members. However, knock-out studies in mice have shown that absence of individual members produce pathologic phenotypes in the intestinal mucosa [21]. For example, specific intestinal knock-out of *cldn7* in mice results in mucosal ulcerations and lethal inflammation despite preservation of colonic tight junctions [32]. A defect in the intestinal permeability of small organic solutes is observed in these mice, allowing bacterial products to permeate the epithelium and cause inflammation.

In the current investigation, an additional complexity of the regulation of key colon tight junction proteins at the level of colon cancer subtypes was revealed. Genomic groups of colon cancers each displayed downregulation of a different set of claudins. The MSI-H/CMS1 group showed downregulation of claudins 1, 3, and 4. The GS/CMS3 group showed downregulation of claudin 1. The mesenchymal CMS4 group displayed downregulation of claudin 2 and 7 and occludin. The CIN/CMS2 group did not show consistent downregulated expression of the examined claudins or occludin. These differences may reflect the diversity of molecular alterations that constitute the landscape of each subtype, leading to activation of carcinogenic cascades with a multitude of direct and indirect downstream targets [33].

Two frequently altered pathways in colon cancer, TGFβ/SMAD and WNT/β-catenin, have been shown to possess clusters of regulatory sites in promoters of claudins 1 to 7 and occludin. In addition, clustered sites of core EMT transcription factors are present in these promoters, suggesting that TGFβ/SMAD and WNT/β-catenin pathways regulate tight junctions, both in a direct and indirect transcription mode. However, a direct correlation of the activation status of the two pathways and expression of individual claudins is not evident. For example, despite activation of the WNT pathway in the majority of CIN and GS colon cancers due to *APC* mutations, claudin expression is highly variable, reflecting putative additional inputs. In contrast to TGFβ/SMAD and WNT/β-catenin pathways, other transcription factors altered in bowel carcinogenesis, such as AP-1, downstream of KRAS/MAPK, MYC, and p53, have a less prominent direct effect in tight junction regulation as they do not possess clustered sites in claudin promoters. The exceptions to these are the promoters of claudin 7 and occludin, which show clustered MYC sites. Despite that, the two proteins are not significantly upregulated in CMS2 cancers where MYC activity is increased, implying a nondominant effect of MYC transcription in these promoters. AP-1-clustered sites are present only in the promoter of claudin 4, but the mRNA of this claudin is not upregulated in CMS3 or CMS1 cancers where the KRAS/MAPK pathway is mostly activated due to activating *KRAS* and *BRAF* mutations, respectively. It should also be mentioned that, besides oncogene mutations, the colon cancer oncogenic cascade components are the subjects of additional post-translational regulations that affect their levels and activity, such as proteasomal degradation [34]. In addition, not all mutations in colon-cancer-associated genes are confirmed oncogenic. Mutations in the most frequently mutated genes in colon cancer, such as *KRAS*, *APC*, *TP53, SMAD4*, *PIK3CA,* and *BRAF*, are known to be oncogenic in almost all the cases. Mutations in other genes, such as *ATM* and epigenetic regulators, are of unknown significance in some cases [35,36]. These observations highlight the complexity of transcriptional regulation of tight junction proteins. The current analysis was unable to capture potential fluctuations of promoter occupancies over time, which could be a source of variability of expression of tight junction proteins during different phases of EMT. This shortcoming is a disadvantage of mRNA genomic analyses in general as these techniques offer only a static overview of mRNA expressions.

Besides their role as key structural components of tight junctions, claudins and occludin have additional roles in regulation of signal transduction, similar to other tetraspanins [37]. Claudin 1 expression is associated with increased activity of the WNT/β-catenin pathway through downregulation of the negative regulator of the pathway E-cadherin [38]. E-cadherin downregulation is mediated by transcription repressor and EMT core factor ZEB1 [39]. An interaction of claudin 1 with Src protein is instrumental in preventing anoikis (loss of adhesion-mediated apoptosis) in cells undergoing EMT [40]. Claudin 1, in collaboration with the nonmembrane tight junction protein ZO-1, interacts with phosphorylated Src and prevents anoikis in parallel with an increased expression of the antiapoptotic protein BCL2, while expression of the other antiapoptotic BCL2 family protein, BCL-xL, was not affected. Loss of the interaction of claudin 1 with Src decreased the effect of claudin 1 overexpression in anoikis prevention [40]. Kinase Src provides inputs for both the PI3K/AKT and MEK/ERK pathways in colonocytes, which may be differentiation state dependent and, as a result, affected by colon cancer cell epithelial or mesenchymal status [41]. KRAS overexpression and MEK activity affect tight junction protein localization in MDCK cells, and MEK inhibition reverses cytoplasmic localization of junction proteins to normal cell membrane positioning [42]. In another experimental system of colorectal cancer cells expressing *BRAF* with V600E mutations, inhibition of the mutant *BRAF* by small kinase inhibitors upregulated claudin 1, which was suppressed in cells with constitutively active mutant *BRAF* [43]. BRAF mutations are predominantly present in the CMS1 subtype of colorectal cancers. In the more prevalent canonical subtype, CMS2, where WNT signaling is upregulated, it has been shown in the current investigation and by others that claudin 1 is upregulated [44]. Thus, colorectal cancer transduction cascades are regulated and provide regulatory signals to tight junctions in a bidirectional manner.

In addition to a structural role in tight junctions, claudin 7 also participates in signal transduction that may mediate a tumor suppression function in colorectal cancer [45]. Claudin 7 inhibition caused loss of extracellular matrix adhesions and increased cell proliferation through loss of the claudin 7 partner integrin β1 and downstream kinase FAK in vitro [46]. In addition, claudin 7 expression is influenced by the activity of the WNT/TCF4 pathway in colonic epithelium and colorectal cancers in a manner that depends on the presence of transcription factor SOX9 [47]. In normal bowel crypts, high WNT/TCF4 in conjunction with SOX9 activity suppresses claudin 7 and keeps residual amounts of the protein restricted in tight junctions promoting cell polarity, while in differentiated cells, low WNT/TCF4 activity is associated with stronger claudin 7 expression. In colorectal cancer cells, WNT/TCF4 activation in the absence of SOX9 leads to high claudin 7 expression, which has diffuse localization in the basolateral membrane and favors loss of polarity. Overexpression of SOX9 in an inducible SOX9 colorectal cancer cell model suppresses claudin 7 expression [47]. *CLDN7* is a p53-regulated gene and is induced by wild-type but not mutant p53 in colorectal cancer [48]. Interestingly, the gene is located at the short arm of chromosome 17 where *TP53* is also located, and the two genes may be codeleted in loss of heterozygosity events. Claudin 7 expression is associated with better prognosis in colorectal cancer patients and induction of epithelial differentiation in colorectal cancer cells [48,49]. The epithelial-specific member of the small GTPase family Rab25 is upregulated in cells with high claudin 7 and contributes to cell trafficking required for cell polarization. Rab25 is a tumor suppressor in the intestine, and when crossed over in apc-deficient mice, it increases the tumor formation frequency [50]. In contrast, well-differentiated colorectal cancer cell lines HT-29 and DLD-1 undergo dedifferentiation upon knockdown of claudin 7 and acquire features of EMT [49]. These data suggest that claudin 7 is a tumor suppressor in colorectal cancer and promotes MET, while its downregulation is associated with reverse EMT.

Efforts to translate the findings of claudin pathophysiology in clinical colorectal cancer have started with the generation of murine monoclonal antibodies against claudin 1 and the discovery of claudin inhibitors [44,51]. A monoclonal antibody blocking claudin 1 was successful in reducing proliferation and survival of colorectal cancer cells in vitro and xenografts in mice in vivo [51]. Moreover, claudin 1 was upregulated in colorectal cancer cells and in tumors in vitro and in vivo following oxaliplatin treatment and development of resistance [52]. A claudin 1-targeting antibody drug conjugate with the antimitotic monomethyl auristatin E (MMAE) as the cytotoxic agent reversed oxaliplatin resistance. A small molecule inhibitor of claudin 1, PDS-0330, was shown to possess antitumor and chemosensitizer activity, at least in part by preventing the interaction of claudin 1 with Src kinase [44]. These results may pave the way for bringing anti-claudin therapies to the clinical arena, where personalized treatments will have to take into consideration the fact that colon cancer subsets differentially express the target molecules. For example, the aforementioned PDS-0330 and other small molecule claudin 1 inhibitors may be more effective in subtypes of colorectal cancers, such as CMS1 and CMS3, which possess lower levels of, and thus potentially more easily inhibitable, claudin 1. On the other hand, a claudin 1-based antibody drug conjugate may be more appropriate for use in CMS2 colon cancers, which express higher levels of the target, thus helping to direct the cytotoxic moiety to the cancer cells. Designing studies that incorporate the targeted molecules as biomarkers of inclusion or response may maximize the chances of successful drug development. In addition, to serve as companion diagnostics, these biomarkers would have to advance through a rigorous analytical and clinical development that will assure their suitability for clinical use.

In conclusion, data presented in this report highlight the heterogeneity of tight junction protein expression and regulation in different genomic subsets of colon cancer. The prominence of the TGFβ/SMAD and WNT/β-catenin pathways as well as EMT core transcriptional regulators in transcriptional regulation of claudins is confirmed based on clustered DNA binding sites on their promoters. A prognostic role for RFS of colon cancer patients according to specific claudin expression has also been documented in genomic subgroups despite not being evident in colon cancer cohorts as a whole. These observations suggest that tight junction proteins could be a target for therapeutic exploitation in carefully selected subsets of colon cancer patients.

## Figures and Tables

**Table 1:** Number of transcription factor binding sites of key transcription factors in colorectal cancer and of core epithelial-to-mesenchymal transition factors in promoters of claudins and occludin.

TF	CLDN1	CLDN2_1	CLDN2_2	CLDN4	CLDN5	CLDN6	CLDN7_1	CLDN7_2	CLDN7_3	CLDN7-4	OCLN_1	OCLN_2
FOS-JUN	1	2	1	5	none	2	1	3	1	1	none	none
SMAD2/3/4	3	4	4	5	9	6	5	9	none	8	3	2
MYC	1	3	1	none	1	3	6	1	8	none	5	1
TCF4	1	4	2	6	4	3	7	2	4	none	6	3
TCF7L2	3	3	2	2	3	2	1	3	none	3	2	3
SOX9	none	1	none	none	none	1	none	2	1	none	1	1
ZEB1	3	5	2	3	5	6	13	6	11	3	7	5
SNAI2	3	6	3	6	2	3	9	2	3	3	7	4
TWIST1	None	3	1	3	none	none	none	none	2	none	1	1
FOXC2	2	1	none	3	none	3	none	3	none	2	3	4
CDX2	none	3	none	2	none	2	none	1	none	none	1	1
TP53	2	2	2	2	1	none	none	1	none	1	1	1

## Data Availability

There are no data available associated with this paper beyond data presented.
